# *N*-(3-Octyl-4-oxo-1,3-thia­zolidin-2-yl­idene)benzamide

**DOI:** 10.1107/S1600536810047884

**Published:** 2010-11-24

**Authors:** Hua-Rong Zhao, Hai-Yan Wang, Xiang-Wu Meng

**Affiliations:** a Department of Chemistry, Zhejiang University, Hangzhou 310027, People’s Republic of China

## Abstract

In the title compound, C_18_H_24_N_2_O_2_S, the thia­zolidinone ring is almost coplanar [maximum atomic deviation = 0.017 (3) Å], and is coplanar with the phenyl ring [dihedral angle = 0.62 (13)°]. The octyl group displays an extended conformation. In the crystal, weak inter­molecular C—H⋯O hydrogen bonds link the mol­ecules into supra­molecular chains along [210].

## Related literature

For pharmaceutical applications of thia­zolidinones, see: Dwivedi *et al.* (1972 ▶); Chandrakant *et al.* (2004 ▶). For the synthesis, see: Peng *et al.* (2004 ▶).
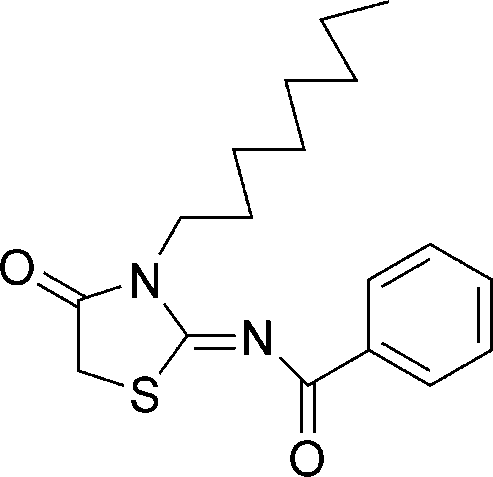


## Experimental

### 

#### Crystal data

C_18_H_24_N_2_O_2_S
*M* *_r_* = 332.45
Triclinic, 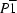

*a* = 5.3342 (3) Å
*b* = 8.6196 (5) Å
*c* = 20.0775 (12) Å
α = 97.008 (5)°
β = 92.870 (4)°
γ = 99.477 (4)°
*V* = 901.41 (9) Å^3^
*Z* = 2
Mo *K*α radiation
μ = 0.19 mm^−1^
*T* = 293 K
0.26 × 0.18 × 0.16 mm

#### Data collection

Oxford Diffraction Nova A diffractometer
8685 measured reflections
3205 independent reflections
2371 reflections with *I* > 2σ(*I*)
*R*_int_ = 0.039

#### Refinement

*R*[*F* ^2^ > 2σ(*F* ^2^)] = 0.051
*wR*(*F* ^2^) = 0.145
*S* = 1.03
3205 reflections
208 parameters
H-atom parameters constrained
Δρ_max_ = 0.18 e Å^−3^
Δρ_min_ = −0.17 e Å^−3^

### 

Data collection: *CrysAlis PRO* (Oxford Diffraction, 2008 ▶); cell refinement: *CrysAlis RED* (Oxford Diffraction, 2008 ▶); data reduction: *CrysAlis RED*; program(s) used to solve structure: *SHELXS97* (Sheldrick, 2008 ▶); program(s) used to refine structure: *SHELXL97* (Sheldrick, 2008 ▶); molecular graphics: *ORTEP-3 for Windows* (Farrugia, 1997 ▶); software used to prepare material for publication: *WinGX* (Farrugia, 1999 ▶).

## Supplementary Material

Crystal structure: contains datablocks I, global. DOI: 10.1107/S1600536810047884/xu5080sup1.cif

Structure factors: contains datablocks I. DOI: 10.1107/S1600536810047884/xu5080Isup2.hkl

Additional supplementary materials:  crystallographic information; 3D view; checkCIF report

## Figures and Tables

**Table 1:** Hydrogen-bond geometry (Å, °)

*D*—H⋯*A*	*D*—H	H⋯*A*	*D*⋯*A*	*D*—H⋯*A*
C4—H4⋯O2^i^	0.93	2.45	3.365 (4)	168

Symmetry code: (i) 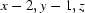.

**Fig. 1.**
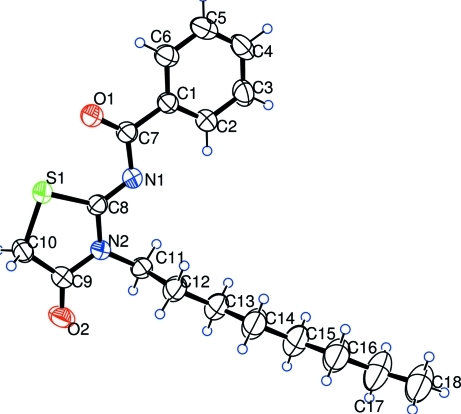
The molecular structure of the title compound with 40% probability displacement ellipsoids.

**Fig. 2.**
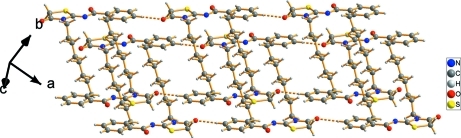
Crystal packing for I viewed down the a axis.

## supplementary crystallographic information

### Comment

Thiazolidinones have broad applications as anticonvulsant (Dwivedi *et al.*, 1972) and anti-microbial drugs (Chandrakant *et al.*, 2004). We report here the structure of a new thiazolidinone derivative, I, Fig. 2.

The thiazolidinyl ring and phenyl ring are almost co-planar with the dihedral angle of 0.67 (0.18)°. In the crystal structure, weak intermolecular C—H···O hydrogen bonds, Table 1, link the molecules to form one-dimensional supra-molecular chains, Fig. 1.

### Experimental

The title compound was prepared followed to the procedure reported by Peng *et al.* (2004). NH_4_SCN (0.152 g, 2 mmol) and [bmim][PF_4_] (2 ml) was mixed in a 50 ml flask equipped with a dropping funnel and then was cooled in an ice-water bath. Next benzoyl chloride(0.284 g, 2 mmol) was added drop by drop and stirred for a further 20 min (disappearance of the raw material was monitored by TLC). n-Octylamine (2 mmol) was then added to the same reaction vessel at room temperature and the mixture was stirred for 20 min more. *N-*benzoyl-*N'-*octylthiourea was formed. After that, ethyl chloroacetate (2.4 mmol) and anhydrous sodium acetate (0.196 g, 2.4 mmol) was added to the flask, and the mixture was heated at 80°C for 2 h. The salts were firstly leached with water (10 ml×2), and the crude product was collected by filtration. Recrystallization from ethanol gave pure product as a yellow crystalline solid.

### Refinement

H atoms were placed in calculated positions with C—H = 0.93-0.97 Å and refined in riding mode with U_iso_(H) = 1.5U_eq_(C) for methyl H atoms and 1.2U_eq_(C) for the others.

### Crystal data

**Table:**

C_18_H_24_N_2_O_2_S	*Z* = 2
*M**_r_* = 332.45	*F*(000) = 356
Triclinic, *P*1	*D*_x_ = 1.225 Mg m^−^^3^
Hall symbol: -p 1	Mo *K*α radiation, λ = 0.71073 Å
*a* = 5.3342 (3) Å	Cell parameters from 2856 reflections
*b* = 8.6196 (5) Å	θ = 4.5–67.0°
*c* = 20.0775 (12) Å	µ = 0.19 mm^−^^1^
α = 97.008 (5)°	*T* = 293 K
β = 92.870 (4)°	Prism, yellow
γ = 99.477 (4)°	0.26 × 0.18 × 0.16 mm
*V* = 901.41 (9) Å^3^	

### Data collection

**Table:**

Oxford Diffraction Nova A diffractometer	2371 reflections with *I* > 2σ(*I*)
Radiation source: fine-focus sealed tube	*R*_int_ = 0.039
graphite	θ_max_ = 25.1°, θ_min_ = 2.1°
ω scans	*h* = −6→5
8685 measured reflections	*k* = −9→10
3205 independent reflections	*l* = −23→23

### Refinement

**Table:**

Refinement on *F*^2^	Primary atom site location: structure-invariant direct methods
Least-squares matrix: full	Secondary atom site location: difference Fourier map
*R*[*F*^2^ > 2σ(*F*^2^)] = 0.051	Hydrogen site location: inferred from neighbouring sites
*wR*(*F*^2^) = 0.145	H-atom parameters constrained
*S* = 1.03	*w* = 1/[σ^2^(*F*_o_^2^) + (0.0633*P*)^2^ + 0.4272*P*] where *P* = (*F*_o_^2^ + 2*F*_c_^2^)/3
3205 reflections	(Δ/σ)_max_ < 0.001
208 parameters	Δρ_max_ = 0.18 e Å^−^^3^
0 restraints	Δρ_min_ = −0.17 e Å^−^^3^

### Special details

**Table:**

Geometry. All e.s.d.'s (except the e.s.d. in the dihedral angle between two l.s. planes) are estimated using the full covariance matrix. The cell e.s.d.'s are taken into account individually in the estimation of e.s.d.'s in distances, angles and torsion angles; correlations between e.s.d.'s in cell parameters are only used when they are defined by crystal symmetry. An approximate (isotropic) treatment of cell e.s.d.'s is used for estimating e.s.d.'s involving l.s. planes.
Refinement. Refinement of *F*^2^ against ALL reflections. The weighted *R*-factor *wR* and goodness of fit *S* are based on *F*^2^, conventional *R*-factors *R* are based on *F*, with *F* set to zero for negative *F*^2^. The threshold expression of *F*^2^ > σ(*F*^2^) is used only for calculating *R*-factors(gt) *etc*. and is not relevant to the choice of reflections for refinement. *R*-factors based on *F*^2^ are statistically about twice as large as those based on *F*, and *R*- factors based on ALL data will be even larger.

### Fractional atomic coordinates and isotropic or equivalent isotropic displacement parameters (Å^2^)

**Table:**

	*x*	*y*	*z*	*U*_iso_*/*U*_eq_	
N2	0.2339 (4)	0.3596 (2)	0.30556 (10)	0.0496 (5)	
N1	−0.1304 (4)	0.2176 (2)	0.34097 (10)	0.0492 (5)	
C9	0.4393 (5)	0.4805 (3)	0.32305 (14)	0.0533 (6)	
C8	0.0680 (5)	0.3283 (3)	0.35492 (12)	0.0464 (6)	
C10	0.4427 (5)	0.5499 (3)	0.39502 (14)	0.0585 (7)	
H10A	0.4422	0.6630	0.3982	0.070*	
H10B	0.5952	0.5337	0.4197	0.070*	
O1	−0.2580 (4)	0.2654 (2)	0.44909 (9)	0.0680 (6)	
O2	0.5957 (4)	0.5200 (2)	0.28388 (11)	0.0733 (6)	
S1	0.16338 (13)	0.45285 (8)	0.42998 (3)	0.0534 (2)	
C7	−0.2882 (5)	0.1901 (3)	0.39283 (13)	0.0508 (6)	
C1	−0.5089 (5)	0.0581 (3)	0.37455 (13)	0.0495 (6)	
C11	0.1963 (5)	0.2715 (3)	0.23775 (13)	0.0569 (7)	
H11A	0.1125	0.1638	0.2402	0.068*	
H11B	0.3613	0.2663	0.2202	0.068*	
C2	−0.5494 (5)	−0.0279 (3)	0.31152 (14)	0.0598 (7)	
H2	−0.4341	−0.0064	0.2792	0.072*	
C6	−0.6820 (5)	0.0229 (3)	0.42264 (15)	0.0606 (7)	
H6	−0.6555	0.0795	0.4656	0.073*	
C5	−0.8909 (6)	−0.0944 (4)	0.40707 (18)	0.0712 (8)	
H5	−1.0049	−0.1172	0.4395	0.085*	
C4	−0.9324 (6)	−0.1783 (4)	0.34373 (19)	0.0735 (9)	
H4	−1.0759	−0.2565	0.3331	0.088*	
C13	−0.0006 (7)	0.2535 (5)	0.12043 (15)	0.0816 (10)	
H13A	0.1634	0.2576	0.1013	0.098*	
H13B	−0.0658	0.1432	0.1241	0.098*	
C12	0.0397 (6)	0.3448 (4)	0.18997 (14)	0.0730 (9)	
H12A	0.1241	0.4523	0.1872	0.088*	
H12B	−0.1249	0.3508	0.2077	0.088*	
C3	−0.7605 (6)	−0.1462 (4)	0.29574 (17)	0.0721 (9)	
H3	−0.7867	−0.2041	0.2530	0.086*	
C15	−0.2276 (8)	0.2222 (6)	0.00389 (18)	0.1053 (13)	
H15A	−0.2838	0.1109	0.0076	0.126*	
H15B	−0.0679	0.2307	−0.0176	0.126*	
C14	−0.1781 (8)	0.3108 (5)	0.07302 (17)	0.0976 (12)	
H14A	−0.3401	0.3090	0.0930	0.117*	
H14B	−0.1106	0.4207	0.0691	0.117*	
C17	−0.4809 (10)	0.1911 (7)	−0.1089 (2)	0.1358 (19)	
H17A	−0.5318	0.0792	−0.1055	0.163*	
H17B	−0.3262	0.2022	−0.1326	0.163*	
C16	−0.4209 (9)	0.2749 (6)	−0.04136 (19)	0.1114 (14)	
H16A	−0.5784	0.2690	−0.0189	0.134*	
H16B	−0.3621	0.3859	−0.0452	0.134*	
C18	−0.6818 (9)	0.2415 (7)	−0.1504 (2)	0.1289 (18)	
H18A	−0.7081	0.1765	−0.1933	0.193*	
H18B	−0.6304	0.3505	−0.1567	0.193*	
H18C	−0.8375	0.2302	−0.1280	0.193*	

### Atomic displacement parameters (Å^2^)

**Table:**

	*U*^11^	*U*^22^	*U*^33^	*U*^12^	*U*^13^	*U*^23^
N2	0.0480 (11)	0.0528 (12)	0.0450 (11)	0.0005 (10)	0.0052 (9)	0.0055 (9)
N1	0.0485 (11)	0.0499 (12)	0.0463 (12)	0.0009 (10)	0.0016 (9)	0.0058 (9)
C9	0.0484 (14)	0.0498 (15)	0.0606 (16)	0.0042 (12)	0.0051 (12)	0.0080 (12)
C8	0.0474 (13)	0.0485 (14)	0.0435 (13)	0.0085 (11)	0.0025 (10)	0.0074 (11)
C10	0.0509 (15)	0.0552 (16)	0.0643 (17)	−0.0023 (12)	−0.0024 (13)	0.0066 (13)
O1	0.0687 (12)	0.0731 (13)	0.0520 (11)	−0.0099 (10)	0.0110 (9)	−0.0052 (10)
O2	0.0613 (12)	0.0734 (14)	0.0804 (14)	−0.0074 (10)	0.0208 (11)	0.0104 (11)
S1	0.0525 (4)	0.0583 (4)	0.0445 (4)	0.0010 (3)	0.0008 (3)	−0.0002 (3)
C7	0.0477 (14)	0.0528 (15)	0.0519 (15)	0.0065 (12)	0.0025 (11)	0.0100 (12)
C1	0.0447 (13)	0.0500 (14)	0.0537 (15)	0.0063 (11)	0.0025 (11)	0.0099 (12)
C11	0.0572 (15)	0.0606 (16)	0.0497 (15)	0.0037 (13)	0.0105 (12)	0.0012 (13)
C2	0.0558 (16)	0.0597 (17)	0.0600 (17)	−0.0015 (13)	0.0014 (13)	0.0091 (14)
C6	0.0517 (15)	0.0645 (18)	0.0671 (18)	0.0083 (13)	0.0082 (13)	0.0153 (14)
C5	0.0541 (17)	0.073 (2)	0.089 (2)	0.0048 (15)	0.0147 (16)	0.0263 (18)
C4	0.0518 (16)	0.0626 (19)	0.102 (3)	−0.0080 (14)	−0.0050 (17)	0.0241 (18)
C13	0.088 (2)	0.097 (3)	0.0558 (18)	0.011 (2)	0.0007 (16)	0.0037 (17)
C12	0.077 (2)	0.086 (2)	0.0543 (17)	0.0132 (17)	0.0032 (15)	0.0036 (16)
C3	0.0703 (19)	0.0631 (19)	0.073 (2)	−0.0051 (15)	−0.0111 (16)	0.0013 (15)
C15	0.116 (3)	0.130 (4)	0.063 (2)	0.014 (3)	−0.010 (2)	0.004 (2)
C14	0.105 (3)	0.122 (3)	0.063 (2)	0.018 (2)	−0.0113 (19)	0.009 (2)
C17	0.153 (4)	0.185 (5)	0.068 (3)	0.049 (4)	−0.022 (3)	−0.004 (3)
C16	0.120 (3)	0.137 (4)	0.073 (2)	0.021 (3)	−0.012 (2)	0.006 (2)
C18	0.129 (4)	0.182 (5)	0.076 (3)	0.042 (4)	−0.017 (3)	0.003 (3)

### Geometric parameters (Å, °)

**Table:**

N2—C9	1.380 (3)	C4—C3	1.384 (5)
N2—C8	1.383 (3)	C4—H4	0.9300
N2—C11	1.463 (3)	C13—C14	1.492 (5)
N1—C8	1.297 (3)	C13—C12	1.504 (4)
N1—C7	1.390 (3)	C13—H13A	0.9700
C9—O2	1.212 (3)	C13—H13B	0.9700
C9—C10	1.493 (4)	C12—H12A	0.9700
C8—S1	1.741 (2)	C12—H12B	0.9700
C10—S1	1.802 (3)	C3—H3	0.9300
C10—H10A	0.9700	C15—C14	1.489 (5)
C10—H10B	0.9700	C15—C16	1.502 (5)
O1—C7	1.221 (3)	C15—H15A	0.9700
C7—C1	1.493 (4)	C15—H15B	0.9700
C1—C2	1.374 (4)	C14—H14A	0.9700
C1—C6	1.395 (4)	C14—H14B	0.9700
C11—C12	1.503 (4)	C17—C16	1.451 (5)
C11—H11A	0.9700	C17—C18	1.477 (6)
C11—H11B	0.9700	C17—H17A	0.9700
C2—C3	1.384 (4)	C17—H17B	0.9700
C2—H2	0.9300	C16—H16A	0.9700
C6—C5	1.371 (4)	C16—H16B	0.9700
C6—H6	0.9300	C18—H18A	0.9600
C5—C4	1.373 (5)	C18—H18B	0.9600
C5—H5	0.9300	C18—H18C	0.9600
			
C9—N2—C8	116.6 (2)	C12—C13—H13A	108.5
C9—N2—C11	120.8 (2)	C14—C13—H13B	108.5
C8—N2—C11	122.6 (2)	C12—C13—H13B	108.5
C8—N1—C7	116.6 (2)	H13A—C13—H13B	107.5
O2—C9—N2	122.6 (3)	C11—C12—C13	113.0 (3)
O2—C9—C10	126.0 (2)	C11—C12—H12A	109.0
N2—C9—C10	111.4 (2)	C13—C12—H12A	109.0
N1—C8—N2	119.3 (2)	C11—C12—H12B	109.0
N1—C8—S1	128.94 (19)	C13—C12—H12B	109.0
N2—C8—S1	111.79 (18)	H12A—C12—H12B	107.8
C9—C10—S1	108.19 (18)	C2—C3—C4	119.8 (3)
C9—C10—H10A	110.1	C2—C3—H3	120.1
S1—C10—H10A	110.1	C4—C3—H3	120.1
C9—C10—H10B	110.1	C14—C15—C16	116.1 (4)
S1—C10—H10B	110.1	C14—C15—H15A	108.3
H10A—C10—H10B	108.4	C16—C15—H15A	108.3
C8—S1—C10	91.98 (12)	C14—C15—H15B	108.3
O1—C7—N1	125.0 (2)	C16—C15—H15B	108.3
O1—C7—C1	120.7 (2)	H15A—C15—H15B	107.4
N1—C7—C1	114.3 (2)	C15—C14—C13	117.1 (4)
C2—C1—C6	119.0 (2)	C15—C14—H14A	108.0
C2—C1—C7	122.1 (2)	C13—C14—H14A	108.0
C6—C1—C7	118.9 (2)	C15—C14—H14B	108.0
N2—C11—C12	113.0 (2)	C13—C14—H14B	108.0
N2—C11—H11A	109.0	H14A—C14—H14B	107.3
C12—C11—H11A	109.0	C16—C17—C18	116.8 (4)
N2—C11—H11B	109.0	C16—C17—H17A	108.1
C12—C11—H11B	109.0	C18—C17—H17A	108.1
H11A—C11—H11B	107.8	C16—C17—H17B	108.1
C1—C2—C3	120.5 (3)	C18—C17—H17B	108.1
C1—C2—H2	119.7	H17A—C17—H17B	107.3
C3—C2—H2	119.7	C17—C16—C15	118.6 (4)
C5—C6—C1	120.5 (3)	C17—C16—H16A	107.7
C5—C6—H6	119.7	C15—C16—H16A	107.7
C1—C6—H6	119.7	C17—C16—H16B	107.7
C6—C5—C4	120.2 (3)	C15—C16—H16B	107.7
C6—C5—H5	119.9	H16A—C16—H16B	107.1
C4—C5—H5	119.9	C17—C18—H18A	109.5
C5—C4—C3	119.9 (3)	C17—C18—H18B	109.5
C5—C4—H4	120.0	H18A—C18—H18B	109.5
C3—C4—H4	120.0	C17—C18—H18C	109.5
C14—C13—C12	115.2 (3)	H18A—C18—H18C	109.5
C14—C13—H13A	108.5	H18B—C18—H18C	109.5

### Hydrogen-bond geometry (Å, °)

**Table:**

*D*—H···*A*	*D*—H	H···*A*	*D*···*A*	*D*—H···*A*
C4—H4···O2^i^	0.93	2.45	3.365 (4)	168

Symmetry codes: (i) *x*−2, *y*−1, *z*.

## References

[bb1] Chandrakant, G. & Gaikwad, N. G. (2004). *Bioorg. Med. Chem.* **12**, 2151–2161. 10.1016/j.bmc.2004.02.024 15080915

[bb2] Dwivedi, C., Gupta, T. K. & Parmar, S. S. (1972). *J. Med. Chem.* **15**, 553–554. 10.1021/jm00275a031 5035284

[bb3] Farrugia, L. J. (1997). *J. Appl. Cryst.* **30**, 565.

[bb4] Farrugia, L. J. (1999). *J. Appl. Cryst.* **32**, 837–838.

[bb5] Oxford Diffraction (2008). *CrysAlis PRO* and *CrysAlis RED* Oxford Diffraction Ltd, Yarnton, England.

[bb6] Peng, Y.-Q., Song, G.-H. & Huang, F.-F. (2004). *J. Chem. Res.* **10**, 676–678.

[bb7] Sheldrick, G. M. (2008). *Acta Cryst.* A**64**, 112–122. 10.1107/S0108767307043930 18156677

